# New genomes, new taxa and deep questions in the eukaryotic tree of life: a meeting report on the EMBO comparative genomics conference

**DOI:** 10.1186/2041-9139-2-22

**Published:** 2011-11-23

**Authors:** Alex de Mendoza, Iñaki Ruiz-Trillo

**Affiliations:** 1 Institut de Biologia Evolutiva (UPF-CSIC), Passeig Marítim de la Barceloneta 37-49, 08003 Barcelona, Spain; 2 Departament de Genètica, Universitat de Barcelona, Av. Diagonal, 645, 08028 Barcelona, Spain; 3 Institució Catalana per a la Recerca i Estudis Avançats (ICREA), Passeig Lluís Companys, 23 08010 Barcelona, Spain

## Abstract

A report on the meeting Comparative Genomics of Eukaryotic Microorganisms: understanding the complexity of diversity. Sant Feliu de Guíxols, Spain. October 15-20, 2011.

## 

'We scientists are like the lovers in 1950s films, absolutely insatiable. After one genome will come another, and another, and we will not stop until we have sequenced all of them' said microbiologist Antonio Lazcano more than a decade ago. The truth is that this year's EMBO conference series on the Comparative Genomics of Eukaryotic Microorganisms has proven Lazcano to be somehow right. Importantly, however, these new genomes are giving us useful insights into eukaryotic diversity, biology and evolution. Even more importantly, thanks to high-throughput sequencing technologies the cost of sequencing is rapidly dropping providing researchers with the opportunity not only to analyze hundreds of genomes but to obtain the first genome data from previously neglected eukaryotic taxa. Below we report some of the highlights of the meeting that both emphasize this trend and are potentially relevant to the readers of *EvoDevo*.

## Fungi, subtelomeric regions and experimental evolution

It is clear that there exists a huge bias in genomic sampling efforts across the diversity of the eukaryotic tree. For example, the Opisthokonta, the clade that includes Fungi and Metazoa, continues to be by far the most well-sampled eukaryotic superclade, whereas genomic data on Excavata and Rhizaria remain scarce. In this meeting more than half of the presentations concerned the analysis of genomes and/or transcriptomes of Fungi, which is not surprising given that more than 200 fungal genomes have been sequenced to date. Such comprehensive genome sampling enables studies at the level of population genomics, some of which are providing important insights into the subtelomeric regions. It is becoming widely accepted that subtelomeric genes show increased rates of duplication and undergo frequent ectopic recombination without the need for meiosis. Thus, these subtelomeric regions may play important roles in the origin of evolutionary innovation and, specifically, in the generation of genetic diversity. Good examples of this, presented by Kevin Verstrepen (VIB Laboratory for Systems Biology, Belgium), are the yeast MAL genes that show interesting patterns of subfunctionalitzation of the resulting duplicates. Another good example is the evolution of variant surface glycoprotein (VSG) genes, which produce an important surface glycoprotein, in the kinetoplastid parasite *Trypanosoma brucei *(Lindsey Plenderleith, University of Glasgow, UK). Moreover, Patrick Keeling (University of British Columbia, Canada) presented comparative genomic analyses of several species of microsporidians, as well as three different strains of Encephalitozoon. Microsporidians have among the smallest eukaryotic genomes as a result of their adaptation to intracellular parasitism. Keeling's analysis revealed higher variation in subtelomeric regions than in the rest of the genome, and a high degree of conservation in synteny and intergenic regions.

Another good reason to study fungi relates to the interesting field of experimental evolution. Sophisticated molecular biology techniques and extensive genomic sampling enables experiments to be conducted across hundreds of generations in lab conditions. Bernard Dujon (Institute Pasteur, France) presented an interesting series of evolutionary experiments designed to unravel how adaptation works. He reported that they had introduced a metabolic gene from *Yarrowia lipolytica *inside *Saccharomyces cerevisiae*, causing a severely unfit phenotype. They subsequently grew this strain for hundreds of generations in non-limiting media, eventually observing an increase in fitness. By genomic re-sequencing they discovered that the insert had been amplified by multiple episomes, which were later re-introduced in tandem repeats inside the genome, allowing an increase in sequence changes. Finally, Jure Piskur (Lund University, Sweden) presented a case of parallel evolution in yeast, in which two distantly related lineages had evolved a convergent lifestyle by re-wiring cis-regulatory motifs of the same metabolic pathway.

## Horizontal gene transfer

A remarkable example of the role that horizontal gene transfer (HGT) plays in eukaryotic evolution was presented by Tom Richards (Natural History Museum London, UK). His group analyzed the role of HGT in the evolution of the osmotrophic lifestyle. Their genome-wide analysis revealed at least 34 HGT events from Fungi to the oomycote *Phytophtora infestants*, which shares a very similar lifestyle with fungi. More importantly, the bulk of transferred genes are involved in the processes of osmotrophy and plant parasitic mechanisms (specifically plant cell breakdown, sugar scavenging, and plant cell effector proteins) suggesting that HGT played a significant role in the convergent evolution towards the osmotrophic capacity of the oomycetes. Interestingly, following the same pipeline they were only able to infer nine ancient transfers between fungi and plants.

## Whole genome duplications

Another important subject that arose during the conference was the importance of whole genome duplications (WGD) in eukaryotes. Some examples, besides plants and vertebrates, are the Saccharomycotina, in which WGD was followed by a rapid loss of duplicates, or the ancestral duplication of Zigomycota, in which Rhizopus suffered a third round of WGD. Analyses of the dynamics of paralog retention and synteny loss shed some light both on sex-chromosome evolution and sensory perception (Luis M. Corrochano, Universidad de Sevilla, Spain; and Ken Wolfe, Trinity College, Ireland).

## Tree of life (TOL)

The tree of life and the efforts to obtain a well-supported tree of eukaryotes was one of the main topics of the conference with four consecutive sessions. Toni Gabaldón (Center for Genomic Regulation, Barcelona) presented a promising automatic method of iterative and nested phylogenetic reconstruction, in which after every node a new dataset of shared genes is re-calculated allowing a scalable resolution at more recent nodes (the tips). The deeper nodes of eukaryotes, however, remain problematic and unsupported. The pipeline allows continuous update as more data become available, so it remains to be seen whether trimming unnecessary taxa and incorporating new genomes from basal nodes will provide better-supported trees. A different strategy was proposed by Céline Brochier-Armanet (Université de Provence, France), who suggested constructing phylogenies with concatenated datasets of operational rather than informational genes. Although operational genes have been considered unsuitable for phylogenies given that they evolve faster, Brochier-Armanet showed a well-supported eukaryote tree inferred using concatenated genes from the midbody toolkit. In her tree, deeper eukaryotic nodes had better resolution than in trees reconstructed from published datasets that are based on informational genes (that is, ribosomal genes). A study to unravel the root of eukaryotes was presented by Ding He (Uppsala University). He showed how they had used a concatenated dataset of 40 nuclear-encoded proteins of bacterial origin (euBacs), mainly of mitochondrial origin, to reconstruct the tree of eukaryotes. The use of euBacs allowed the tree to be rooted using alpha-proteobacteria as an out-group. The topology showed that Discoba (a group of excavates that comprises Heterolobosea, Jakobids and Euglenozoa) is the sister-group to the remaining eukaryotes. Then Andrew J. Roger (Dalhousie University, Canada) nicely introduced us to the long-standing problem of long branch attraction as well as other systematic errors and the possible solutions for overcoming them. He emphasized the use of improved phylogenetic models and improved taxonomic sampling. For example, he showed how the addition of several slowly evolving excavates radically changed the topology on the deeper nodes of the eukaryotic tree. For this reason it was good to learn of the first jakobid (*Andalucia godoyi*) to be fully sequenced, by Marek Elias (University of Ostrava, Czech Republic). This is particularly important if Discoba really are the most basal eukaryotic group. Elias presented a very compelling argument for why it is worth sequencing previously neglected taxa such as Andalucia or other jakobids. Within this same clade (Discoba), Lillian Fritz-Laylin (University of California, US) showed how the genome of *Naegleria gruberi *has changed our views of the last common ancestor of Eukaryotes and presented promising results on the development of molecular techniques for this organism.

Two talks discussed work on diatoms, which are ecologically relevant microorganisms. Andrew Allen (J. Craig Venter Institute, US) reported the presence of genes involved in the urea cycle in the genomes of both *Thalassiosira pseudonana *and *Phaeodactylum tricornutum*. These genes had previously been found only in metazoans and prokaryotes. Chris Bowler (Institut de Biologie de l'Ecole normale supériere, France) presented the epigenomic map of DNA methylation in the diatom *P. tricornutum*. Bowler reported a set of convincing experiments that clearly show that methylation is found preferentially on transposable elements, such that these are silenced by default. Transposable elements are later unsilenced allowing an increase in evolutionary rates, when the organism faces stressful conditions.

## Mitochondrial genomes and metagenomics

Two successful symposiums dealt with mitochondrial genomes and metagenomics. In the first one, Gertraud Burger (University of Montreal, Canada) and Michael W. Gray (Dalhousie University, Canada) presented examples of degenerate mitochondrial genomes among diplonemids and euglenids. The mitochondrial genomes of diplonemids demonstrate an extremely complex behavior, with all genes being partitioned among hundreds of small chromosomes. These fragments are transcribed and trans-spliced, as well as heavily edited, making the adaptive value of this process a big mystery. All this seems to be led by some kind of 'guiding RNAs', the nature of which has been investigated in a series by Burger.

It is probably the field of metagenomics that best emphasizes the role that high-throughput sequencing technologies are playing in shaping this, as well as other, disciplines. Patrick Wincker (Genoscope, France) gave an overview of the ambitious Tara Oceans project, the goal of which is to explore global ocean biodiversity and ecosystems with the use of high-throughput sequencing, imaging methods and modeling tools. A recurrent technical issue throughout the talk was the importance of the single-celled amplified genomes in order to better understand the main players of marine ecological environments. Finally, Alexandra Z. Worden (Monterey Bay Aquarium Research Institute, US) focused on eukaryotic phytoplankton, pinpointing the importance of linking organism and gene function in order to increase our current understanding of marine ecosystems.

Given the enormous amount of genomic data that is currently being generated, Lazcano's statement certainly bears some truth. However, one wonders whether in the future, and with the limits of data processing, we will have to think carefully about which organisms it will be worth obtaining the genome from. Nevertheless, we should enjoy and celebrate the power that technology is providing us to understand the diversity of eukaryotes in unforeseen ways.

## Competing interests

The authors declare that they have no competing interests.

## Authors' contributions

Both AdM and IRT wrote the manuscript. Both authors read and approved the final manuscript.

